# Cannabinoid Receptor CB2 Is Involved in Tetrahydrocannabinol-Induced Anti-Inflammation against Lipopolysaccharide in MG-63 Cells

**DOI:** 10.1155/2015/362126

**Published:** 2015-01-14

**Authors:** Lei Yang, Fei-Fei Li, Yu-Chen Han, Bin Jia, Yin Ding

**Affiliations:** ^1^State Key Laboratory of Military Stomatology, Department of Orthodontics, School of Stomatology, Fourth Military Medical University, Xi'an, Shaanxi 710032, China; ^2^Department of Stomatology, The 264th Hospital of PLA, Taiyuan, Shanxi 030001, China; ^3^Department of Orthopaedics, Xijing Hospital, Fourth Military Medical University, Xi'an, Shaanxi 710032, China

## Abstract

Cannabinoid Δ9-tetrahydrocannabinol (THC) is effective in treating osteoarthritis (OA), and the mechanism, however, is still elusive. Activation of cannabinoid receptor CB2 reduces inflammation; whether the activation CB2 is involved in THC-induced therapeutic action for OA is still unknown. Cofilin-1 is a cytoskeleton protein, participating in the inflammation of OA. In this study, MG-63 cells, an osteosarcoma cell-line, were exposed to lipopolysaccharide (LPS) to mimic the inflammation of OA. We hypothesized that the activation of CB2 is involved in THC-induced anti-inflammation in the MG-63 cells exposed to LPS, and the anti-inflammation is mediated by cofilin-1. We found that THC suppressed the release of proinflammatory factors, including tumor necrosis factor *α* (TNF-*α*), interleukin- (IL-) 1*β*, IL-6, and IL-8, decreased nuclear factor-*κ*B (NF-*κ*B) expression, and inhibited the upregulation of cofilin-1 protein in the LPS-stimulated MG-63 cells. However, administration of CB2 receptor antagonist or the CB2-siRNA, not CB1 antagonist AM251, partially abolished the THC-induced anti-inflammatory effects above. In addition, overexpression of cofilin-1 significantly reversed the THC-induced anti-inflammatory effects in MG-63 cells. These results suggested that CB2 is involved in the THC-induced anti-inflammation in LPS-stimulated MG-63 cells, and the anti-inflammation may be mediated by cofilin-1.

## 1. Introduction

Oversecretion of proinflammatory factors from osteoblasts plays vital roles in the progress of osteoarthritis [1, 2], and high levels of proinflammatory factors in bones and joints induce pain, cartilage loss, and even joint dysfunction [3, 4]. Therefore, reducing the release of proinflammatory factors from osteoblasts is an effective therapy for OA. Traditional anti-inflammatory drugs, including steroidal anti-inflammatory drugs and nonsteroid anti-inflammatory drugs (NSAID), are effective in alleviating the symptoms of OA [5, 6]. However, long-term use of these drugs brings about many side effects, such as gastritis, gastric ulcer, and/or immune suppression [5, 7]. Therefore, a novel anti-inflammatory drug with mild side effects is needed urgently in the treatment of OA.

Cannabinoid Δ9-tetrahydrocannabinol (THC) is a main bioactive component from marijuana [8], and THC is effective in treating OA [9]; however, the mechanism is still not clear. THC exerts bioactivities by activating cannabinoid receptors [10]. At present, two main cannabinoid receptors have been discovered, including CB1 and CB2. Activation of CB1 receptor is associated predominantly with a dampening down of neuronal excitability, whereas activation of CB2 receptors is related to reducing immune cell function, including decreases of proinflammatory factors release [11]. In addition, CB2 receptor is expressed in osteoarthritis joints. Therefore, we aimed at CB2 receptor, not CB1, as one of the research targets in this study. Cofilin is a cytoskeleton protein family, including nonmuscle cofilin (cofilin-1), muscle cofilin (cofilin-2), and actin depolymerizing factor (ADF). Cofilin-1 and ADF are primarily expressed in nonmuscle mammalian cells, and cofilin-1 predominates [12]. Moreover, cofilin-1 is associated with many inflammatory conditions, such as OA and inflammatory pain [13, 14], and some investigations indicated that overexpression of cofilin-1 can decrease glucocorticoid receptor expression and NF-*κ*B activity [15, 16]. However, whether cofilin-1 is involved in the therapeutic effects of THC in treating OA is still unclear.

MG-63 cell is an osteosarcoma cell-line, used widely to mimic osteoblasts* in vitro* [17]. Lipopolysaccharide (LPS) is a component of the outer membrane of gram-negative bacteria, which has been used mainly as a proinflammatory substance in several investigations [18]. And MG-63 cells exposed to LPS can be used as an inflammatory model of OA* in vitro* [19].

In this study, we hypothesized that cannabinoid CB2 receptor is involved in THC-induced anti-inflammation in LPS-stimulated MG-63 cells, and the THC-induced anti-inflammation may be mediated by cofilin-1 protein.

## 2. Materials and Methods

### 2.1. Cell Culture and Reagents

MG-63, an osteogenic human osteosarcoma cell-line, was obtained from the America Type Culture Collection (ATCC). Cells were cultured in Minimum Essential Medium Eagle with Earl's salts (MEM) medium (Hyclone, USA) containing 10% fetal bovine serum (Hyclone, USA), 100 U/mL penicillin, and 0.1 mg/mL streptomycin in a humidified atmosphere containing 5% CO_2_ and 95% air at 37°C. THC, LPS, and doxycycline were purchased from Sigma-Aldrich (St. Louis, MO, USA). CB2 antagonist AM630 and CB1 antagonist AM251 were obtained from Tocris Bioscience (UK). Anti-CB2 antibody and anti-cofilin-1 antibody were purchased from the Abcam (Cambridge, UK).

### 2.2. Experimental Protocols

**Figure 1:**
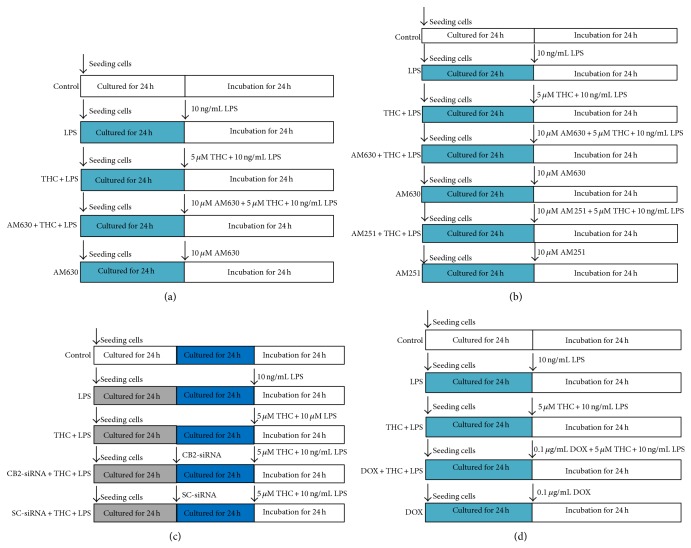
Experimental protocol diagram. (a) The MG-63 cells were assigned into five groups, including control, LPS, THC + LPS, AM630 + THC + LPS, and AM630 groups. After the treatments for 24 h, immunocytochemistry and western blot were taken to investigate the expression of CB2 receptor. (b) The MG-63 cells were assigned into seven groups as shown in this figure. After the treatments, ELISA was used to evaluate the inflammatory factors concentrations in the supernatants. (c) The MG-63 cells were assigned into five groups, including control, LPS, THC + LPS, CB2-siRNA + THC + LPS, and SC-siRNA + THC + AM630 groups. After the treatments, western blot was taken to investigate the expression of CB2 receptor and ELISA was used to assess the inflammatory factors release. (d) The cells were divided into control, LPS, THC + LPS, doxycycline (DOX) + THC + LPS, and DOX groups, western blot was taken to investigate the expression of cofilin-1 protein, and ELISA was taken to evaluate the concentrations of proinflammatory factors in the supernatants.

The MG-63 cells were divided into five groups, including control, LPS, THC + LPS, AM630 + THC + LPS, and AM630 alone groups. After the treatments, immunocytochemistry staining and western blot were used to investigate the expression of CB2 receptor in the cells (Figure 1(a)). Then, the cells were assigned into seven groups, including control, LPS, THC + LPS, AM630 + THC + LPS, AM630 alone, AM251 + THC + LPS, and AM251 alone groups. After the treatments, inflammatory factors levels in the supernatants were evaluated by enzyme-linked immunosorbent assay (ELISA) kits (Figure 1(b)). To further determine the role of CB2 in the THC-induced anti-inflammatory effects in MG-63 cells, the cells were divided into five groups, including control, LPS, THC + LPS, CB2-siRNA + THC + LPS, and scrambled- (SC-) siRNA + THC + LPS groups, after the treatments as shown in this figure, and western blot and ELISA kits were used to evaluate NF-*κ*B expression and inflammatory factors release, respectively (Figure 1(c)). Then the MG-63 cells were divided into five groups, including control, LPS, THC + LPS, doxycycline (DOX) + THC + LPS, and DOX alone groups, after the treatments, and western blot and ELISA kits were used to evaluate cofilin-1 expression and inflammatory factors release, respectively (Figure 1(d)).

### 2.3. Enzyme-Linked Immunosorbent Assay (ELISA)

The release of TNF-*α*, IL-1*β*, IL-6, and IL-8 from the LPS-stimulated MG-63 cells was determined by the corresponding ELISA kit (R & D Systems, Minneapolis, MN, USA) according to the instructions. The MG-63 cells were seeded in a 24-well plate at a density of 5 × 10^4^ cells/well. After the incubation for 24 h, the supernatants were collected and centrifuged, and then the cell-free supernatants were used for ELISA assay.

### 2.4. Immunofluorescence Staining

The MG-63 cells were seeded into five confocal microscopy special dishes at a density of 2 × 10^4^ cells/dish. After the treatments, the MG-63 cells were washed three times with phosphate buffer solution (PBS) and then fixed with 4% paraformaldehyde solution for 30 min and blocked with 50 mg bovine serum albumin (BSA)/mL in PBS for 30 min. Cells were incubated with primary anti-CB2 antibody (1 : 50) overnight at 4°C and then washed three times with PBS, before incubation with Cy3-labeled secondary antibody (1 : 200, Beyotime, China) for 1 h at room temperature. The 4′,6-diamidino-2-phenylindole (DAPI) staining solution (200 *μ*L) was added into each dish for 5 min, and then the dishes were washed three times with PBS. CB2 expression was observed by a laser scanning confocal microscope (FV10i, Olympus, Japan) (Excitation = 550 nm, Emission = 570 nm).

### 2.5. Western Blot

After the treatments, the MG-63 cells were washed twice with ice-cold PBS (pH 7.2) and harvested with ice-cold PBS and then centrifuged at 14000 ×g for 5 min at 4°C. Nuclear and cytosolic extracts were prepared using a Nuclear and Cytoplasmic Protein Extraction Kit (Beyotime, China) according to the manufacturer's instructions. The protein concentration was determined by using the Bradford assay. The samples were fractionated on 10% SDS-polyacrylamide gel electrophoresis (SDS-PAGE) and transferred to polyvinylidene fluoride membranes (Chemicon International, Millipore, Billerica, MA, USA). The membranes were blocked for 1 h at room temperature with 5% nonfat milk in PBS-Tween 20 (0.1%) and then incubated with different primary antibodies (CB2, 1 : 200; NF-*κ*B, 1 : 2000; cofilin, 1 : 2000). The membrane was incubated with electrochemiluminescence reagent for 10 min and exposed to X-ray film. The signals were quantified by densitometry using a western blotting detection system (Alpha Innotech, USA). *β*-actin and GAPDH (Cowin Inc. China) served as the loading control.

### 2.6. CB2 Small Interfering RNA

MG-63 cells were transfected with specific CB2 targeting small interfering RNA (siRNA) designed to knockdown CB2 protein expression (Dharmacon, USA). Briefly, MG-63 cells (70% confluent) were transfected with 100 pmol CB2-siRNA or scrambled- (SC-) siRNA using LipofectAMINE 2000 (Invitrogen) according to the manufacturer's protocol. After the transfection for 24 h, the cells were then treated with different drugs and assessed.

### 2.7. Statistical Analysis

The results of this study were expressed as means ± SD, which were obtained from at least three independent experiments for each condition. The statistical significance of the results was evaluated with one-way variance analysis (one-way ANOVA), followed by Tukey's multiple comparison test. *P* < 0.05 was taken to indicate statistical significance.

## 3. Results

### 3.1. THC Attenuated LPS-Induced Inflammation in MG-63 Cells

**Figure 2:**
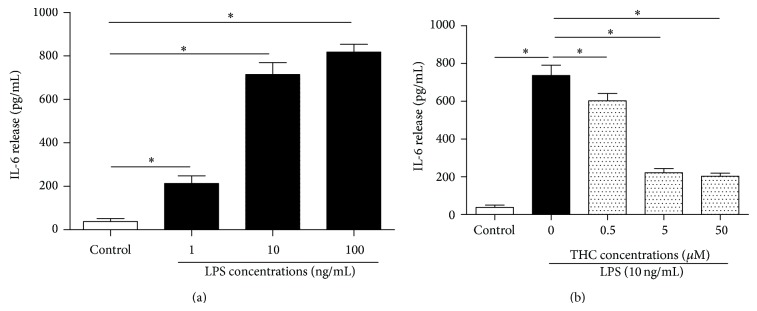
THC decreased LPS-induced IL-6 release from MG-63 cells dose-dependently. (a) MG-63 cells were treated with 0, 1, 10, and 100 ng/mL LPS for 24 h. (b) The MG-63 cells were treated with different concentrations of THC plus 10 ng/mL LPS for 24 h. IL-6 release was determined by ELISA kits. Results are means ± SD (*n* = 8).  ^*^: *P* < 0.05.

The MG-63 cells were exposed to different concentrations of LPS for 24 h (Figure 2(a)), causing a dose-dependent release of IL-6 from the cells. IL-6 is a proinflammatory factor, which was evaluated as an index reflecting the inflammation level of the LPS-treated MG-36 cells. The presence of 10 ng/mL LPS for 24 h increased the IL-6 level to 714.2 ± 55.4 pg/mL, compared with 38.5 ± 13.2 pg/mL of the control (*P* < 0.05) in the supernatants. We used 10 ng/mL LPS in the subsequent experiments. Coadministration of 0.5, 5, and 50 *μ*M THC significantly reduced the IL-6 release from the LPS-stimulated cells (Figure 2(b)), suggesting that THC induced an anti-inflammation. We used 5 *μ*M THC in the subsequent experiments.

### 3.2. THC Upregulated the Expression of CB2 Receptor in MG-63 Cells

**Figure 3:**
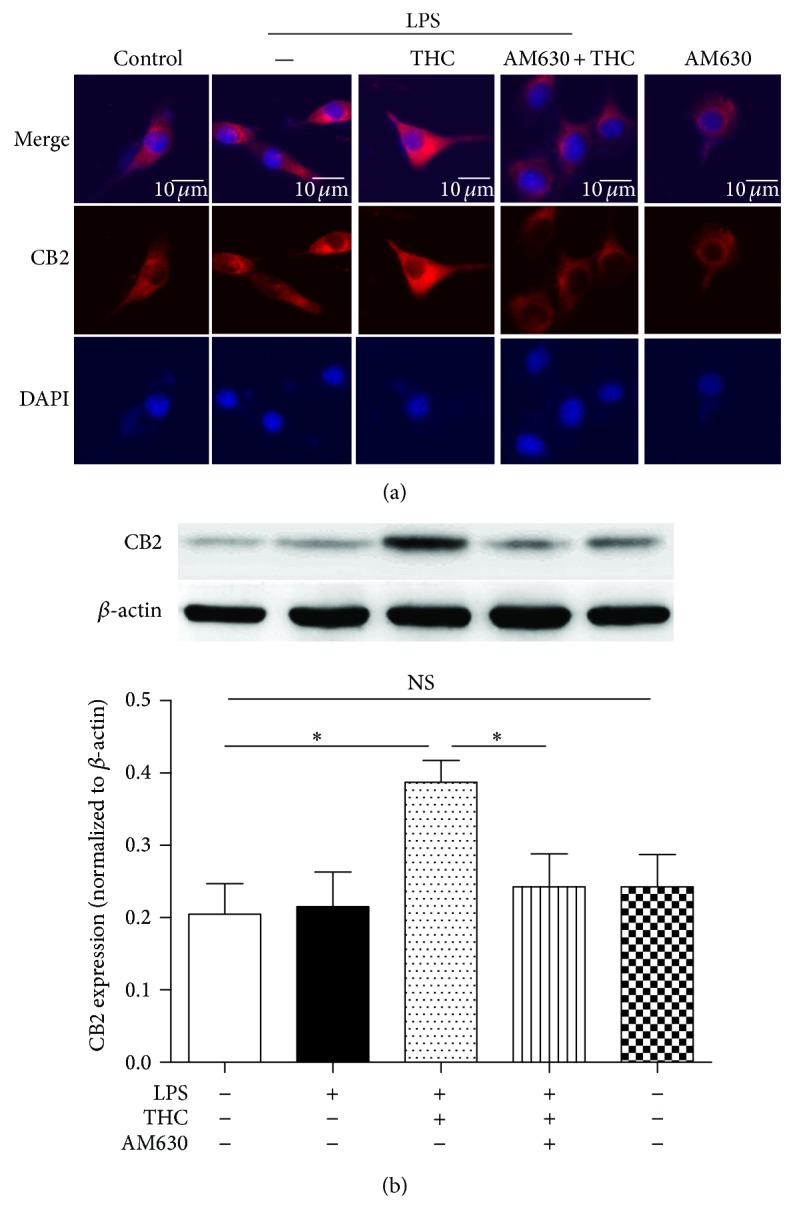
THC upregulated CB2 receptor expression. Immunofluorescent staining (a) and western blot (b) were used to determine the CB2 protein expression. The cells were treated with or without 10 ng/mL LPS for 24 h in the presence or absence of 5 *μ*M THC or 10 *μ*M AM630 (CB2 receptor antagonist). The CB2 receptor expression (red) was observed by a confocal microscope, and stronger signal indicated higher expression of CB2 protein. Nuclei were counter-stained with DAPI (blue). Bar = 10 *μ*m. Results are means ± SD (*n* = 6).  ^*^: *P* < 0.05; NS: no significance.

Immunocytochemistry and western blot were used to determine the expression of CB2 protein in MG63 cells. We observed that LPS alone did not induce a marked change of CB2 protein expression (Figures 3(a)-3(b)), and THC (5 *μ*M) upregulated CB2 protein expression. However, coadministration of CB2 antagonist AM630 (10 *μ*M) significantly inhibited the THC-induced upregulation of CB2 expression and did not induce a marked change of CB2 expression alone.

### 3.3. CB2, Not CB1, Was Involved in THC-Induced Reduction of Proinflammatory Factors Release and NF-*κ*B Expression

**Figure 4:**
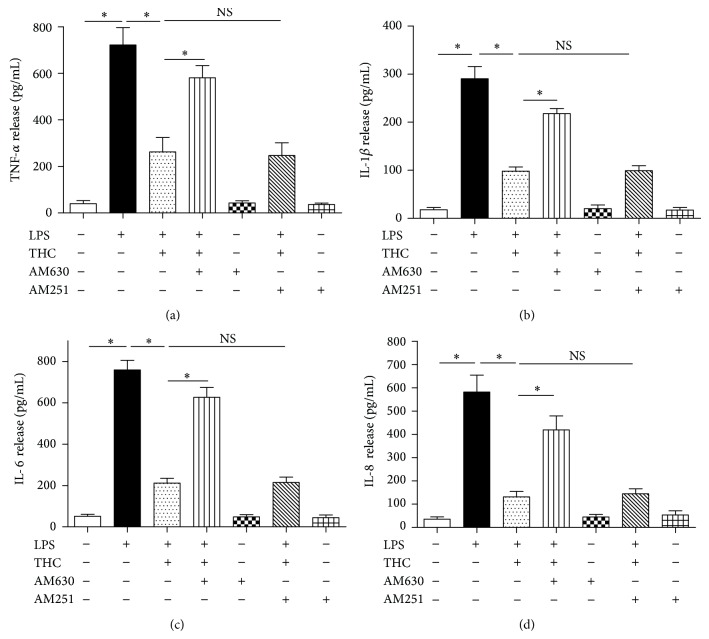
THC decreased the release of TNF-*α*, IL-1*β*, IL-6, and IL-8 from LPS-stimulated MG-63 cells. TNF-*α* (a), IL-1*β* (b), IL-6 (c), and IL-8 (d) release were determined by ELISA kits. THC (5 *μ*M) decreased 10 ng/mL LPS-induced release of TNF-*α*, IL-1*β*, IL-6, and IL-8, and CB2 antagonist AM630 (10 *μ*M) reversed these effects. Results are means ± SD (*n* = 6).  ^*^: *P* < 0.05; NS: no significance.

**Figure 5:**
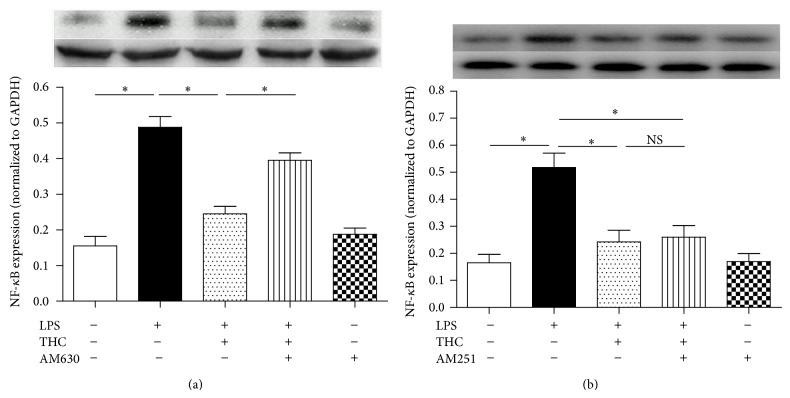
THC reduced NF-*κ*B expression via CB2 receptor in LPS-stimulated MG-63 cells. NF-*κ*B expression was assessed by western blot. THC reduced LPS-induced upregulation of NF-*κ*B expression, and CB2 antagonist AM630 (a), not CB1 antagonist AM251 (b), partially abolished this effect. Results are means ± SD, (*n* = 6).  ^*^: *P* < 0.05; NS: no significance.

Incubation with LPS (10 ng/mL) for 24 h sharply increased the concentrations of TNF-*α*, IL-1*β*, IL-6, and IL-8 in the supernatants (*P* < 0.05) and the upregulation of NF-*κ*B expression in MG-63 cells, and coadministration of THC (5 *μ*M) significantly reduced the release of the proinflammatory factors (Figures 4(a)–4(d)) and the upregulation of NF-*κ*B (Figures 5(a)-5(b)). However, CB2 antagonist AM630, not CB1 antagonist AM251, inhibited the THC-induced reduction of release of proinflammatory factors and NF-*κ*B expression, indicating that the THC-induced anti-inflammatory effects may be mediated by CB2 receptor, not by CB1.

### 3.4. CB2-siRNA Reversed THC-Induced Anti-Inflammatory Effects

**Figure 6:**
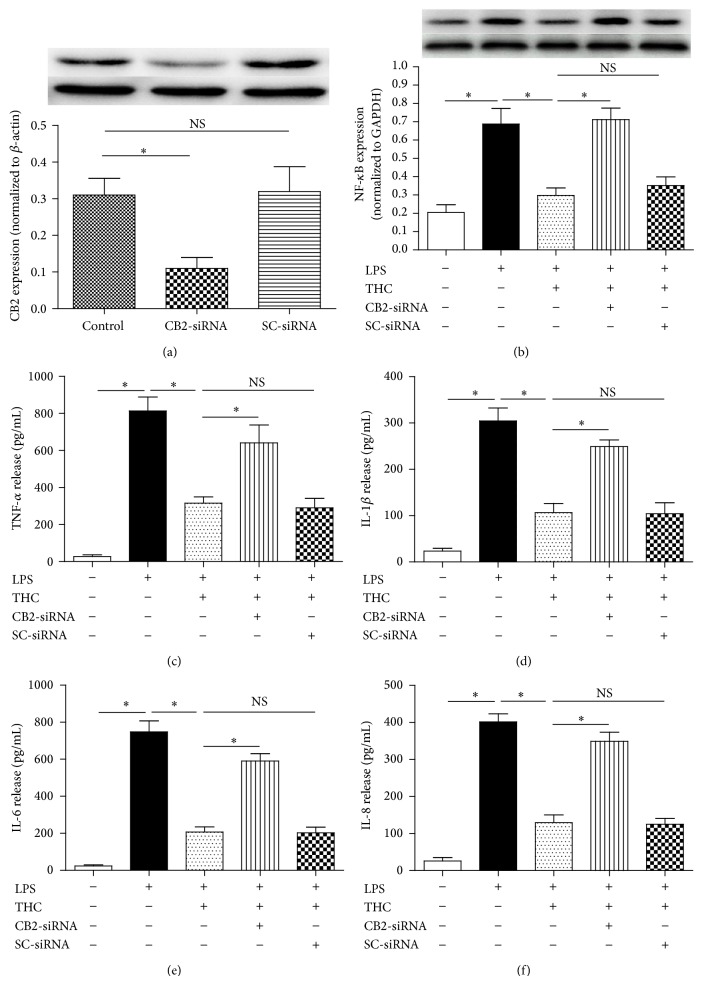
CB2-siRNA reversed THC-induced decrease of inflammatory factors release. MG-63 cells were assigned into five groups, control (no LPS, THC, or siRNA): cultured in drug-free medium; LPS: cells exposed to 10 ng/mL LPS for 24 h; THC + LPS: cells exposed to 5 *μ*M THC plus 10 ng/mL LPS for 24 h; CB2-siRNA + THC + LPS: cells incubated with CB2-siRNA for 24 h and then exposed to 5 *μ*M THC plus 10 ng/mL LPS for 24 h; scrambled siRNA (SC-siRNA) + THC + LPS: cells incubated with SC-siRNA for 24 h and then exposed to 5 *μ*M THC plus 10 ng/mL LPS for 24 h. (a) CB2-siRNA significantly downregulated the expression of CB2 receptor, assessed by western blot (*n* = 3). (b) CB2-siRNA significantly reversed THC-induced effect on NF-*κ*B expression (*n* = 6). (c)–(f) CB2-siRNA significantly reversed THC-induced effects on the release of TNF-*α*, IL-1*β*, IL-6, and IL-8 (*n* = 6). Results are means ± SD.  ^*^: *P* < 0.05; NS: no significance.

To further determine whether the THC-induced anti-inflammation was mediated by CB2 receptor in MG-63 cells, we used CB2-siRNA to knockdown the CB2 protein expression in MG-63 cells. We found that CB2-siRNA was effective in reducing the expression of CB2 (Figure 6(a)). In addition, CB2-siRNA reversed the THC-induced inhibition of NF-*κ*B expression (Figure 6(b)) and proinflammatory factors release (Figures 6(c)–6(f)), including TNF-*α*, IL-1*β*, IL-6, and IL-8 (*P* < 0.05).

### 3.5. Cofilin-1 Was Involved in the THC-Induced Anti-Inflammatory Effect in MG-63 Cells

Incubation with 10 ng/mL LPS for 24 h markedly increased the cofilin-1 expression in MG-63 cells (*P* < 0.05) compared with the cells cultured in drug-free medium, and coadministration of 5 *μ*M THC inhibited the cofilin-1 expression significantly (Figure 6(a)). CB2 antagonist AM630 partially reversed the THC-induced inhibition of cofilin-1 expression.

In order to further determine the role of cofilin-1 in THC-induced anti-inflammation in the MG-63 cells, we used doxycycline, which can upregulate the expression of cofilin-1 significantly* in vitro* [20, 21]. We observed that 0.1 *μ*g/mL doxycycline markedly increased the expression of cofilin-1 in MG-63 cells (Figure 6(b)). And coadministration of 0.1 *μ*g/mL doxycycline partially reversed THC-induced decrease of IL-6 and TNF-*α* release (Figures 6(c)-6(d)). These findings indicated that cofilin-1 was involved in the THC-induced anti-inflammation via CB2 in the MG-63 cells exposed to LPS.

## 4. Discussion

**Figure 7:**
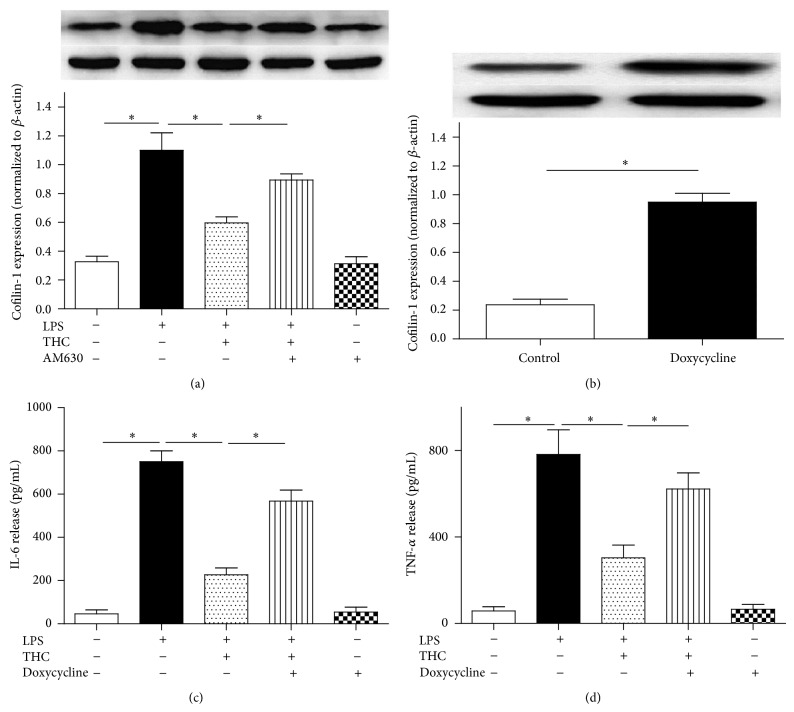
The upregulation of cofilin-1 inhibited the THC-induced anti-inflammation. (a) CB2 antagonist reversed the THC-induced effect on cofilin-1 expression. (b) Doxycycline induced an overexpression of cofilin-1 in the MG-63 cells. (c) Doxycycline abolished THC-induced reduction of IL-6 release. (d) Doxycycline abolished the THC-induced reduction of TNF-*α* release. The cells were treated with different drugs for 24 h (LPS: 10 ng/mL; THC: 5 *μ*M; AM630: 10 *μ*M; doxycycline: 0.1 *μ*g/mL), western blot was performed to evaluate the cofilin-1 expression, and ELISA was used to test the concentrations of IL-6 and TNF-*α* in the supernatants. Results are means ± SD (*n* = 6).  ^*^: *P* < 0.05.

In this study, we found that the presence of 5 *μ*M THC attenuated the inflammation in MG-63 cells exposed to 10 ng/mL LPS. Meanwhile THC increased CB2 protein expression and inhibited the upregulation of NF-*κ*B and cofilin-1 expression in the MG-63 cells. However, administration of CB2 antagonist AM630 or CB2-siRNA, not CB1 antagonist AM251, partially reversed the THC-induced anti-inflammatory effects. Moreover, upregulation of cofilin-1 protein partially abolished the THC-induced anti-inflammation (Figure 7). These findings indicated that CB2 receptor is involved in the THC-induced anti-inflammation in the MG-63 cells exposed to LPS, and the anti-inflammation may be mediated by cofilin-1 protein.

OA is one of the most common causes of disability in elder adults, especially in the population aged over 65 years. Oversecretion of proinflammatory cytokines, including TNF-*α*, IL-6, and IL-1*β*, contributes to the severity and the progression of OA [3]. High levels of proinflammatory cytokines in bones and joints can damage articular cartilage by inducing chondrocyte apoptosis and proteoglycan depletion [22]. Therefore, inhibiting the secretion of proinflammatory cytokines is vital to prevent the progression of cartilage degeneration at the initial stage of OA [5]. At present, corticosteroids and NSAID are used widely to attenuate the inflammation of OA [23, 24]. As these drugs just relieve the symptoms of OA rather than cure the disease, side effects, including vomiting, gastritis, and immune suppression, emerged with repeated and long-term use [5, 25]. Thus, a drug that can treat OA with slight and less side effects is needed urgently.

THC is main cannabinoid compounds from marijuana, which has a number of therapeutic applications, including anti-inflammation and analgesic effects [26]. However, THC has rarely been well studied in treating OA and its therapeutic mechanism is still not clear. In most cases, THC is used as a painkiller, which relieves pain via activating cannabinoid and opioid receptors expressed in the brain [27]. Therefore, researchers paid more attention to the CNS system rather than the peripheral tissue during investigating the mechanism of THC in anti-inflammation. In this study, we found that THC attenuated LPS-induced proinflammatory factors release from the MG-63 cells, including TNF-*α*, IL-1*β*, IL-6, and IL-8, and THC reduced the upregulation of NF-*κ*B expression in the MG-63 cells, indicating that the THC-induced anti-inflammatory effects may be associated with its anti-inflammation. Moreover, selective CB2 receptor antagonist AM630 or CB2-siRNA, not CB1 antagonist AM251, significantly reversed the THC-induced anti-inflammatory effects and the inhibition of NF-*κ*B expression, suggesting that the THC-induced anti-inflammation may be mediated by CB2 receptor in MG-63 cells. As one of the key molecules regulating the release of various inflammatory factors, the expression level of NF-*κ*B reflects the inflammation degree in cells. NF-*κ*B locates in the nucleus, and CB2 is mainly expressed in the cell membrane and cytoplasm [28, 29]; thus we infer that THC may activate CB2 receptors expressed in the cell membrane and cytoplasm of MG-63 cells and then inhibit NF-*κ*B expression to reduce the inflammatory factors release from the MG-63 cells exposed to LPS. Similarly, Gondim et al. reported that CB2 is involved in electroacupuncture- (EA-) induced anti-inflammatory effects in a rat model of arthritis [30]. And Zarruk et al. reported that the activation of microglial CB2 receptor brings about neuroprotection against brain ischemic injury by decreasing the proinflammatory cytokines release in mice [31]. Although THC is a cannabinoid ligand for both CB1 and CB2 receptors, Newton and Klein described that THC downregulates serum IgE levels through CB2 receptor rather than CB1 receptor in an IgE induction model in mice [32]. These findings supported that activation of CB2 receptor may be involved in THC-induced anti-inflammation in MG-63 cells.

Cofilin is a cytoskeleton protein family, which is known to promote actin depolymerization and maintain the stability of cell structure [33]. A study indicated that overexpression of cofilin can induce glucocorticoid resistance [15], and another study mentioned that JWH015, a selective CB2 agonist, modulated the activity of cofilin [34]. Cofilin is also an important signaling molecule for the cytoskeletal and migratory properties of trabecular meshwork cells [34]. The cofilin protein family consists of three isoforms, including cofilin-1, cofilin-2, and ADF. Cofilin-1 and ADF are mainly expressed in non-muscle tissue, and cofilin-1 predominates. Chiang et al. reported that cofilin-1 is involved in isoflurane- (an inhaled anesthetic) induced anti-inflammatory effect [14]; besides CB2 could be activated by isoflurane [35], suggesting that cofilin-1 may be involved in the modulation of inflammation, which might be related to CB2 receptor. Therefore, we investigated cofilin-1 in THC-induced anti-inflammatory effects in this study. We observed that CB2 antagonist AM630 reversed the THC-induced modulation of cofilin-1 expression in the LPS-stimulated MG-63 cells, and overexpression of cofilin-1 abolished the THC-induced anti-inflammation in the cells, indicating that cofilin-1 may mediate the THC-induced anti-inflammatory effects via CB2 receptor. Moreover, Rom et al. reported that CB2 selective agonists GP1a and AM1241 increase the phosphorylation of cofilin in brain tissue, causing protection of blood-brain barrier against inflammatory leukocyte [36]. Therefore, we infer that THC-induced anti-inflammation may be related to the phosphorylation of cofilin in MG-63 cells exposed to LPS. However, how does THC modulate expression or phosphorylation of cofilin-1 protein? Further investigation is needed to answer this question.

There are still some limitations in our investigation. First, our study is* in vitro*; thus the results of this investigation should be further identified in* in vivo* study and clinical trials. Second, the MG-63 cells used here are an osteosarcoma cell-line, not primary osteoblasts, so the results of this study should be accepted cautiously. Third, we just upregulated the cofilin-1 expression in this study; how THC works is still unknown when cofilin-1 is downregulated.

## 5. Conclusions

Our study indicates that cannabinoid CB2 receptor is involved in THC-induced anti-inflammation in MG-63 cells exposed to LPS, and the anti-inflammatory effects may be mediated by cofilin-1 protein.
